# Randomized clinical study of injectable dextrin-based hydrogel as a carrier of a synthetic bone substitute

**DOI:** 10.1007/s00784-023-04868-9

**Published:** 2023-01-28

**Authors:** Alexandra Machado, Isabel Pereira, Filomena Costa, Ana Brandão, José Eduardo Pereira, Ana Colette Maurício, José Domingos Santos, Inês Amaro, Rui Falacho, Rui Coelho, Nuno Cruz, Miguel Gama

**Affiliations:** 1 CEB, Centre of Biological Engineering, University of Minho, Campus de Gualtar, 4710-057 Braga, Portugal; 2 LABBELS, Associate Laboratory, Braga, Guimarães Portugal; 3 Biosckin, Molecular and Cell Therapies S.A., TecMaia, Rua Engenheiro Frederico Ulrich 2650, 4470-605 Maia, Portugal; 4 CECAV, Animal and Veterinary Research Centre, University of Trás-os-Montes and Alto Douro, 5001-801 Vila Real, Portugal; 5 Department of Veterinary Sciences, University of Trás-os-Montes and Alto Douro, 5001-801 Vila Real, Portugal; 6 Departamento de Clínicas Veterinárias, Instituto de Ciências Biomédicas de Abel Salazar (ICBAS), Universidade do Porto (UP), Rua de Jorge Viterbo Ferreira, N° 228, 4050-313 Porto, Portugal; 7 Centro de Estudos de Ciência Animal (CECA), Instituto de Ciências, Tecnologias e Agroambiente da Universidade do Porto (ICETA), Rua D. Manuel II, Apartado 55142, 4051-401 Porto, Portugal; 8 REQUIMTE/LAQV, Departamento de Engenharia Metalúrgica e Materiais, Faculdade de Engenharia, Universidade do Porto, Rua Dr. Roberto Frias, 4200-495 Porto, Portugal; 9 Institute of Integrated Clinical Practice, Faculty of Medicine, University of Coimbra, 3004-504 Coimbra, Portugal; 10 Institute of Oral Implantology and Prosthodontics, Faculty of Medicine, University of Coimbra, 3004-504 Coimbra, Portugal; 11 RESDEVMED, Unipessoal Lda., Travessa do Navega, 436 C, 3885-183 Ovar, Portugal; 12 Faculty of Dentistry, Universitat Internacional de Catalunya, 08017 Barcelona, Spain

**Keywords:** Synthetic bone, Dextrin, Injectable hydrogel, Alveolar preservation

## Abstract

**Objectives:**

This study aimed to improve the performance and mode of administration of a glass-reinforced hydroxyapatite synthetic bone substitute, Bonelike by Biosckin® (BL®), by association with a dextrin-based hydrogel, DEXGEL, to achieve an injectable and moldable device named DEXGEL Bone.

**Methods:**

Twelve participants requiring pre-molar tooth extraction and implant placement were enrolled in this study. BL® granules (250–500 µm) were administered to 6 randomized participants whereas the other 6 received DEXGEL Bone. After 6 months, a bone biopsy of the grafted area was collected for histological and histomorphometric evaluation, prior to implant placement. The performance of DEXGEL Bone and BL® treatments on alveolar preservation were further analyzed by computed tomography and Hounsfield density analysis. Primary implant stability was analyzed by implant stability coefficient technique.

**Results:**

The healing of defects was free of any local or systemic complications. Both treatments showed good osseointegration with no signs of adverse reaction. DEXGEL Bone exhibited increased granule resorption (*p* = 0.029) accompanied by a tendency for more new bone ingrowth (although not statistically significant) compared to the BL® group. The addition of DEXGEL to BL® granules did not compromise bone volume or density, being even beneficial for implant primary stability (*p* = 0.017).

**Conclusions:**

The hydrogel-reinforced biomaterial exhibited an easier handling, a better defect filling, and benefits in implant stability.

**Clinical relevance:**

This study validates DEXGEL Bone safety and performance as an injectable carrier of granular bone substitutes for alveolar ridge preservation.

**Trial registration:**

European Databank on Medical Devices (EUDAMED) No. CIV-PT-18–01-02,705; Registo Nacional de Estudos Clínicos, RNEC, No. 30122.

**Supplementary Information:**

The online version contains supplementary material available at 10.1007/s00784-023-04868-9.

## Introduction

Post-extraction bone remodeling is an inevitable natural phenomenon and can lead to significant ridge dimensional changes with loss of height and width of the alveolar bone [1]. Placing space-maintaining grafts in the edentulous site at the time of extraction is a common approach to prevent or minimize impairment of the supporting structures of an implant [2]. Bone regeneration procedures are essential to successfully settling an implant afterwards, restoring the site with satisfactory functionality and aesthetics. The design of shape fitting hydrogels (HGs) is a recent trend intended to circumvent the poor cohesivity and injectability that restrain bone substitutes use as bone fillers [3–5], conferring suitability for minimal invasive procedures.

DEXGEL is an in situ gelling hydrogel (WO/2011/070529A2) [6] with oxidized dextrin as the base component. Dextrin is a low-cost, broadly available raw material derived from starch, widely used in many industrial applications and accepted as a generally-recognized-as-safe (GRAS) food ingredient [7]. Dextrin is available in medical grade, has high solubility in water and DMSO, and holds hydroxyl groups suitable for bioconjugation, which has prompted this glucose polymer for several biomedical applications [8], including as hydrogels [9–11]. In this work, dextrin was firstly oxidized (ODEX) with sodium periodate to bear aldehyde groups suitable to cross-link with adipic acid dihydrazide (ADH) amine groups, without any chemical initiator that could be potentially harmful for any agents to be embedded within it [12]. ODEX and ADH are spontaneously stitched together upon contact by Schiff base reaction, creating hydrazone bonds (R_2_ C = NNR_2_) [13]. Hydrazone linkages are reversible in water [14], gradually dismantling the hydrogel network as the free and bound polymer chains reach for constant equilibrium. These dynamic covalent bonds endow HGs with injectability and moldability, acquiring a versatile array of shapes for a better defect filling.

The polysaccharide nature of DEXGEL mimics the extracellular matrix of native bone, offering excellent biocompatibility. We have previously reported DEXGEL suitability as a carrier of nanogels, cells, biomolecules, and granular ceramics [12, 15–18]. Moreover, biocompatibility, safety, and effectiveness were assessed through a battery and a combination of in vitro and in vivo approaches. DEXGEL has demonstrated in vitro cyto- and genocompatibility [12, 19]. In vivo biocompatibility and safety were demonstrated through the assessment of the inflammatory response in rat subcutaneous implants [16], subacute systemic toxicity, and skin sensitization using rodent models [18]. The effectiveness of the DEXGEL combined with BL® granules was demonstrated in two different bone defects: in goat critical-sized bone defects [17] and tibial fractures [18]. After extensive pre-clinic evaluation and formulation design, DEXGEL is ready for the first clinical assessment as an injectable and moldable carrier of BL® granules.

Bone graft synthetic substitutes are desired to overcome the limited source and significant morbidity associated with the harvesting of autologous bone grafts [20, 21]. BL®, property of Biosckin, Molecular and Cell Therapies, S.A. (WO2010021559A1) [22], is a synthetic bone graft designed to mimic the inorganic composition of bone. It has been a subject of extensive characterization and clinical evaluation, which provided the conformity of the material for human use [23–30]. Particularly, BL® granules (250 to 500 μm) have been used successfully in maxillofacial surgery to repair bone defects caused by removal of cysts and elevation of the maxillary sinus, resulting in partial regeneration of the bone defect with no adverse reaction [30], and recently functioned as a space filler in appendicular bone defects and maxillary/mandibular bone defects, promoting a faster bone fusion in 14 animals [31]. Although currently unavailable, this study intends to accelerate BL® (250 to 500 μm) market re-entry, now as an innovative injectable device.

This clinical study aimed to improve the performance and mode of administration of the glass-reinforced hydroxyapatite (HA) synthetic bone substitute, BL®, by association with dextrin-based hydrogel, DEXGEL, in the management of alveolar bone. Biocompatibility and bone ingrowth are crucial parameters to be examined for clinical acceptability. Herein, we report a study that aims to validate the clinical safety and efficacy of DEXGEL Bone (DEXGEL + BL®) for the treatment of bone defects through the following analysis: (a) assess the capacity and performance of DEXGEL both as a bioceramic vehicle and an adjuvant matrix in the bone regeneration process, (b) assess the volume and quality of regenerated bone, (c) assess the primary stability of the dental implant, and (d) assess granule stabilization and ease of clinical use. DEXGEL Bone will be applied as an intermediate alveolar regeneration procedure from tooth extraction moment to implant placement, aiming at achieving a volume of bone formed after 6 months equal to the initial post-extraction alveolar volume — primary endpoint.

## Materials and methods

### Study design and overall clinical procedures

**Fig. 1:**
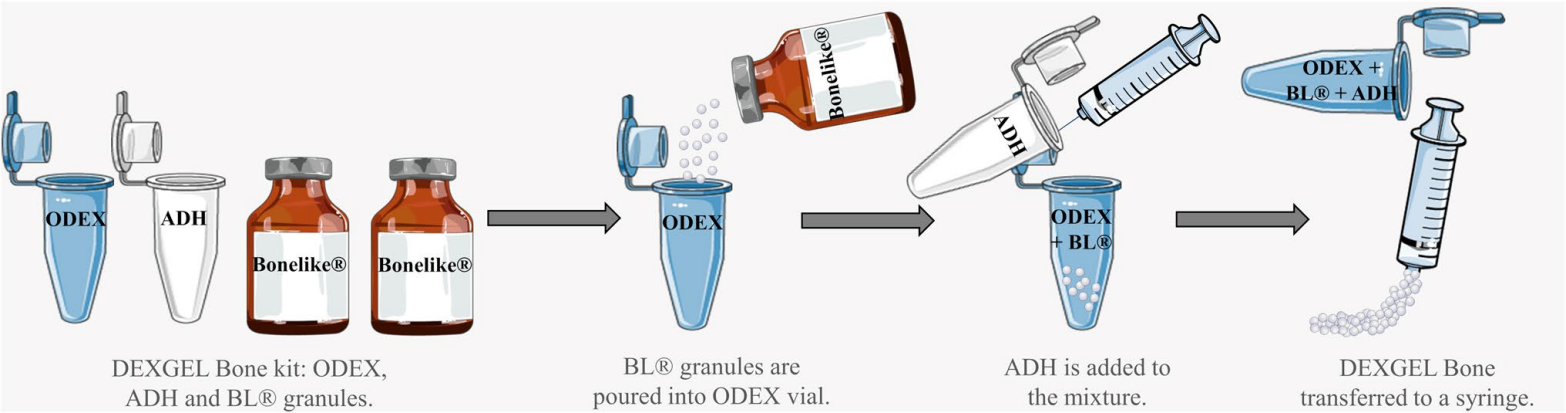
DEXGEL Bone preparation procedure

G*Power 3.1.9.2 software was used a priori to define sample size (Fig. 1 SI). This pilot study was designed for a two-tailed analysis with Student’s *t*-test or Wilcoxon signed rank test (depending on the normality of the results), with a confidence interval (statistical power) of 95% (1 − *β* = 0.95), with a probability of occurrence of a type 1 error of 5% (*α* = 0.05) and assuming an *effect size dz* of 2. The analysis determined that a total of 6 participants for each group were needed to meet aforementioned parameters for the primary endpoint of this study: the volume of bone formed after 6 months equals to the initial post-extraction alveolar volume. All of the participants underwent tooth extraction and socket preservation, with test and control group materials (parallel group design), for 6 months before implant placement.

The overall clinical study involved seven visits to the study facilities by each patient, as listed in Table 1. Postoperative clinical evaluations were performed after 3, 10, and 24 days to determine the presence of any complications such as infection, inflammation, wound dehiscence, or loss of graft material. After 6 months, clinical examinations were performed following implant placement. Afterwards, patients were still closely followed by the dentist over an extended period, however, with no relevance to this paper.

**Table 1:** Overall study procedures listed by order of accomplishment

Medical visit	Main procedures
1st recruitment interview	The candidate is invited to participate in the study; the anamnesis, inclusion, and exclusion criteria are reviewed; and if the candidate is eligible to participate, the information leaflet and declaration of consent are signed
2nd pre-surgical visit	Computed tomography (CT). This visit could be done on the same day as the candidate’s recruitment interview or the same day as the surgical visit
3rd surgical intervention	Surgery for tooth extraction, fill the alveolar socket with medical device and closure of the surgical wound
4th first check-up visit	3 days post-surgery: check-up for visual examination of the extraction site and tissue condition
5th second check-up visit	1 week after first check-up visit: check-up for visual examination of the extraction site and tissue condition, and for suture removal
6th third check-up visit	2 weeks after second check-up visit: check-up for visual examination of the extraction site and tissue condition
7th pre-surgical visit	6 months post-surgery: CT examination. This visit could be done few days before or on the same day as the surgical re-entry
8th surgical re-entry	6 months post-surgery: collect bone sample and evaluate tissue condition, following implant placement

### Recruitment

The recruitment process began with the dissemination of the clinical study within Hospital da Luz, Coimbra. The selection of potential candidates was carried out based on patients with teeth with surgical indication for orthodontic treatment or with unviable teeth for recovery or reconstruction, or patients wishing to rehabilitate a lost dental piece by placing an implant in the upper premolars area. Patients who meet these criteria were informed about the clinical study. Those who showed interest in participating in the study were redirected to a recruitment interview. During the interview, disease diagnosis and inclusion and exclusion criteria were reviewed, and if the participant was eligible to participate, the information leaflet and the declaration of consent were signed. Specific inclusion and exclusion criteria are listed in Table 2. Twelve male and female adults (above 18 years) were assigned into two groups (Table 3), allocated 6 to each group through a simple randomization methodology, according to the date of acceptance of the patients informed declaration of consent.

**Table 2:** Inclusion and exclusion criteria

Inclusion criteria	Exclusion criteria
✓ Individuals requiring tooth extraction and dental implant replacement, in the upper premolars area ✓ Skeletally mature individuals, aged between 18 and 65 ✓ Good general health	✗ Individuals with premolars diagnosed with pulp necrosis and chronic endodontic and/or periodontal pathology involving changes in the alveolar bone (except teeth with endodontic pathology without symptoms of inflammatory and/or bacterial origin) ✗ Accidental avulsion ✗ Individuals with acute or chronic infections, local or distant from the area to be submitted to surgery ✗ Women who are pregnant, breastfeeding, or intending to become pregnant during the study ✗ Individuals with smoking, alcoholic habits, or consumption of illegal substances ✗ Individuals with medical contraindications (severe kidney disease, malignant tumors, uncontrolled diabetes, vascular or neurological damage, bone or metabolic diseases, patients with prosthetic valves, and immunocompromised individuals) ✗ Individuals who are engaged to other clinical study or are still covered by a clinical study insurance ✗ Individuals who demonstrate inability to follow up during the clinical study period

**Table 3:** List of participants

Patient	1	2	3	4	5	6	7	8	9	10	11	12
Sex	M	F	F	F	F	M	M	M	F	F	F	M
Age	40^a^	41^b^	66^c^	43^a^	40^b^	48^b^	41^b^	48^b^	51^b^	47^a^	41^b^	48^b^
Tooth	24	24	14	25	14	15	14	14	24	14	24	25
Group	DEXGEL Bone (test)						BL® (control)					

*F* female, *M* male, *BL®* Bonelike by Biosckin®

^a^Caries

^b^Tooth/root fracture

^c^Extraction due to periodontal reason

### Materials

All reagents used were of the highest degree of purity commercially available, suitable for biopharmaceutical production. Dextrin from Tackidex B 167 (Batch E8747) was kindly provided by Roquette (Lestrem, France), sodium *m‐*periodate (CAS no. 7790‐28‐5) and diethylene glycol (CAS no. 111‐46‐6) were purchased from BIOCHEM Chemopharma (Cosne sur loire, France). Adipic acid dihydrazide (ADH; CAS no. 1071‐93‐8) was supplied by Merck KGaA (Darmstadt, Germany) and endotoxin free phosphate‐buffered saline (PBS; CAS no. 10049‐21‐5) by BioConcept (Allschwil, Switzerland). BL® was provided by Biosckin, Molecular and Cell Therapies, S.A. (Maia, Portugal).

### Preparation of DEXGEL

Dextrin oxidation was performed as previously described [32]. Briefly, aqueous solutions of dextrin (2% w/v) were oxidized with sodium *m*‐periodate (NaIO_4_), to yield the theoretical degree of oxidation of 40%, at room temperature, with stirring, in the dark. The oxidation reaction was stopped after 20 h by dropwise addition of an equimolar amount of diethylene glycol to reduce any unreacted periodate. Sodium *m*‐periodate and diethylene glycol were removed by dialysis, using a 1000-Da cutoff membrane (Merck Millipore, Billerica, MA, USA), and then freeze-dried. ODEX was dissolved in PBS solution (30% w/v) and sterilized by gamma irradiation (IONISOS, Dagneux, France), using a ^60^Co source, at 20 kGy (2 kGy/h), at room temperature. ADH was also dissolved in PBS solution (3.76% w/v) and sterilized by filtration, using a 0.22-μm pore filter membrane (Pall Corporation, Ann Arbor, MI, USA). For the crosslinking reaction, ODEX and ADH solutions were mixed in a 7:3 volume ratio, respectively. ODEX and ADH were packaged in separate microtubes, vacuum sealed, and stored at 4 °C. Three samples of each were analyzed for endotoxin content by Biogerm, S.A. (Moreira, Portugal) and sterility by Sagilab Laboratório de Análises Técnicas, S.A. Heat-resistant laboratory materials were sterilized by autoclave, and heat-sensitive materials were sterilized by ethylene oxide. The sterilization process and subsequent sterility validation were performed according to the ISO 11137:2006 [33] and ISO 11737: 2009 [34] requirements, respectively.

### Preparation of BL® granules

BL® was ready in previous commercially available packages. The production procedure has been reported [22, 35]. Briefly, phase pure HA was prepared by the precipitation between calcium hydroxide [Ca(OH)_2_] (Prolabo, Paris, France) and orthophosphoric acid 85 wt% [H_3_PO_4_] (Merck, Darmstadt, Germany). Filtered and dried HA precipitate was ground into a fine powder, with a granulometry less than 75 mm. A P_2_O_5_–CaO-based glass with the composition of 65P_2_O_5_–15CaO–10CaF_2_10Na_2_O (mol%) was obtained by mixing the following reagent-grade chemicals: calcium hydrogen phosphate dehydrate (CaHPO_4_ · 2H_2_O; Sigma, St. Louis), disodium carbonate (Na_2_CO_3_; Panreac, Spain), calcium fluoride (CaF_2_; Merck, Darmstadt, Germany), and phosphorus pentoxide (P_2_O_5_; Panreac, Spain). A frit was obtained at 1450 °C for 30 min in a platinum crucible. Spherical granules were obtained by mixing 2.5 wt% of glass HA with a pore forming agent, via a dry process, at a rate up to 100 rpm. Then, the mixture was hydrated with purified water and submitted to malaxation. The resulting moist paste was extruded with an extrusion screen of 1 mm (Caleva Extruder 20, Caleva Process Solutions, Blandford, UK) and spheronized (Caleva Spheronizer 120, Caleva Process Solutions, Blandford, UK), and the pellets were then sintered at 1300 °C. Standard sieving techniques were used to obtain the 250–500-µm particle size ranges, displaying an interconnective microporosity structure. BL® was sterilized by gamma irradiation at 25 kGy in Centro de Higienização por Ionização de Produtos S.A., Instituto Tecnológico e Nuclear (Lisboa, Portugal). Table 4 shows a summary of the BL® composition.

**Table 4:** Composition of BL®

Material	Ca/P ratio	HA (%)	α-TCP (%)	β-TCP (%)	Ions	Granule size (μm)	Surface area (m^2^/g)	Porous size (µm)
BL®	1.70	81	17	< 2	Ca^2+^; PO_4_^3−^	250–500	0.368	0.7402–100.35

*Ca/P* calcium/phosphate, *HA* hydroxyapatite, *TCP* tricalcium phosphate, *BL®* Bonelike by Biosckin®

### DEXGEL Bone kit

DEXGEL Bone is an injectable, porous, and osteoconductive bone substitute composed of two phases: (i) dextrin hydrogel matrix, DEXGEL — polymeric phase and (ii) BL®, composed mostly of hydroxyapatite, having a percentage of tricalcium phosphate — ceramic phase. The DEXGEL Bone kit is composed of (i) four vials: one with the ODEX solution, another with ADH (both dissolved in PBS) and 2 vials of BL® with 0.5 g each; (ii) a syringe with needle, to transfer ADH solution and subsequent mixing of all components; and (iii) a second syringe to apply the final formulation into the alveolar socket. Components must be mixed at the time of surgery by the medical team, according to a specific protocol (Fig. 1). Each device was prepared for a final volume of 2 cm^3^, including 0.4 mL of ADH, 0.933 mL of ODEX, and 1 g of BL®. BL® granules, 50% v/v of the total volume of the final formulation (HG), were mixed with the ODEX solution, and the reticulation was achieved adding the ADH solution prior to surgery (Fig. 1), in a proportion of 7:3 (ODEX:ADH) volume ratio. DEXGEL Bone was ready for administration in a pre-gelled moldable form 5 min upon ADH addition.

**Fig. 2:**
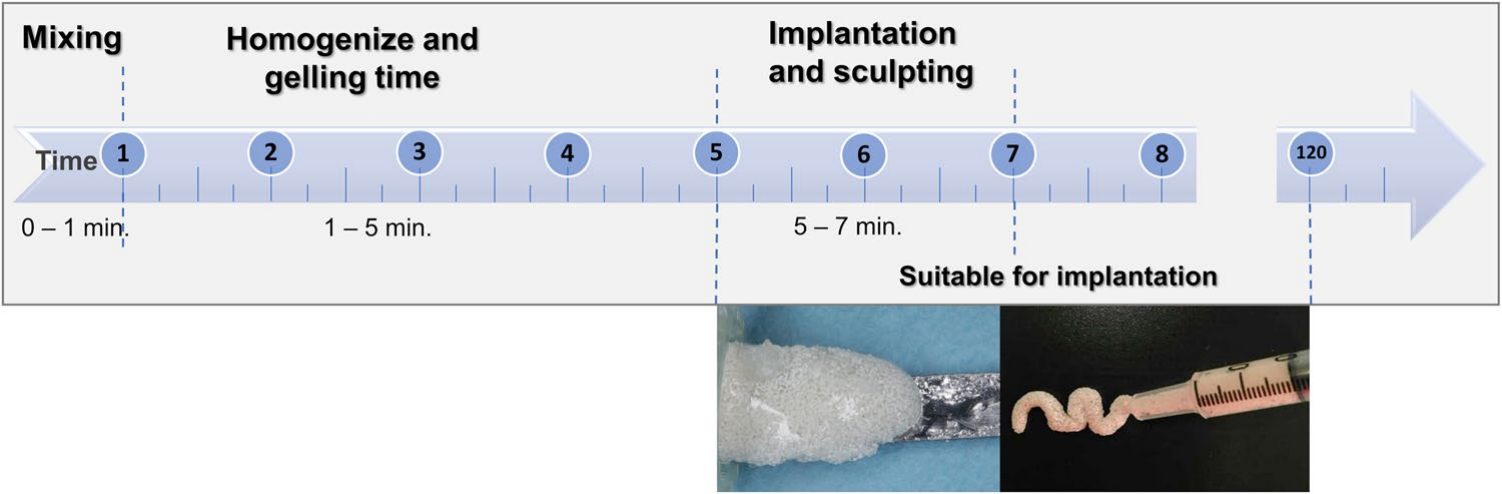
Timeline for DEXGEL Bone preparation and handling

Figure 2 shows the necessary time for device handling at the time of surgery, from components mixing to implantation. The preparation was performed by a technician the moment surgeon began the extraction process. Exodontia generally took 5 to 10 min, which matched the time needed for device preparation. After properly gelled in the form of a moldable paste, DEXGEL Bone could be readily implanted or either rest for an extended period of 2 h before implantation, as most convenient to the surgeon. Within this period, the hydrogel matrix did not dehydrate, thus not compromising granule stabilization. Sculpting could be performed from 1 to 2 min. There was no need for a set time within the defect as in other grafting materials, the gum tissue can therefore be closed right after sculpting.

### Ethical considerations

iBone Therapies (European Databank on Medical Devices, EUDAMED, No. CIV-PT-18–01-02,705; Protocol No. EC.01.01.17) was previously approved by the Portuguese National Authority of Medicines and Health Products, I.P. (INFARMED), after being submitted in the National Registry of Clinical Studies (RNEC, No. 30122), by the National Ethics Commission for Clinical Research (CEIC, RNEC No. 30121) and National Commission for the Protection of Data (CNPD). The production of DEXGEL was carried out in a clean room with ISO class 4 classification, suitable for the production of medical devices, at the facilities of RESDEVMED Unipessoal Lda (Ovar, Portugal). Qualified candidates were provided all the necessary clarifications so that they could make an informed consciousness decision. Twelve participants provided written informed consent. All participants were covered by a civil liability insurance from Haftpflichtverband der Deutschen Industrie (HDI) company. This study followed CONSORT 2010 statement guidelines for reporting parallel group randomized trials.

### Surgical procedure

#### Tooth extraction and biomaterial implantation

The surgical intervention was performed by a dentist with experience in oral surgery and implantology at Hospital da Luz Coimbra (Coimbra, Portugal). After CT and X-ray examination, the patient was anesthetized with Artinibsa (articaine + epinephrine at a dosage of 72 mg/1.8 mL + 0.009 mg/1.8 mL, Inibsa Laboratories, Barcelona, Spain). The indicated tooth was extracted, and alveolar curettage was performed for complete removal of injured tissue and tooth remains. DEXGEL Bone was applied using a syringe and sculpted with a spatula, filling the alveolar socket without exceeding alveolar crest. BL® was mixed with autologous blood previously extracted from the alveolar defect and applied with a spatula. Gum tissue was then sutured to end the process. Volunteers were prescribed with ibuprofen 600 mg (Brufen 600, Mylan, Lda., Lisboa, Portugal) from 12 to 12 h for 3 days and amoxicillin 1 g (Cipamox, Laboratórios Vitória, Amadora, Portugal) from 12 to 12 h for 8 days. In case of allergy, azithromycin 500 mg (Zithromax, Pfizer, NY, USA) was prescribed once daily for 3 days.

#### Sample collection and implant placement

**Fig. 3:**
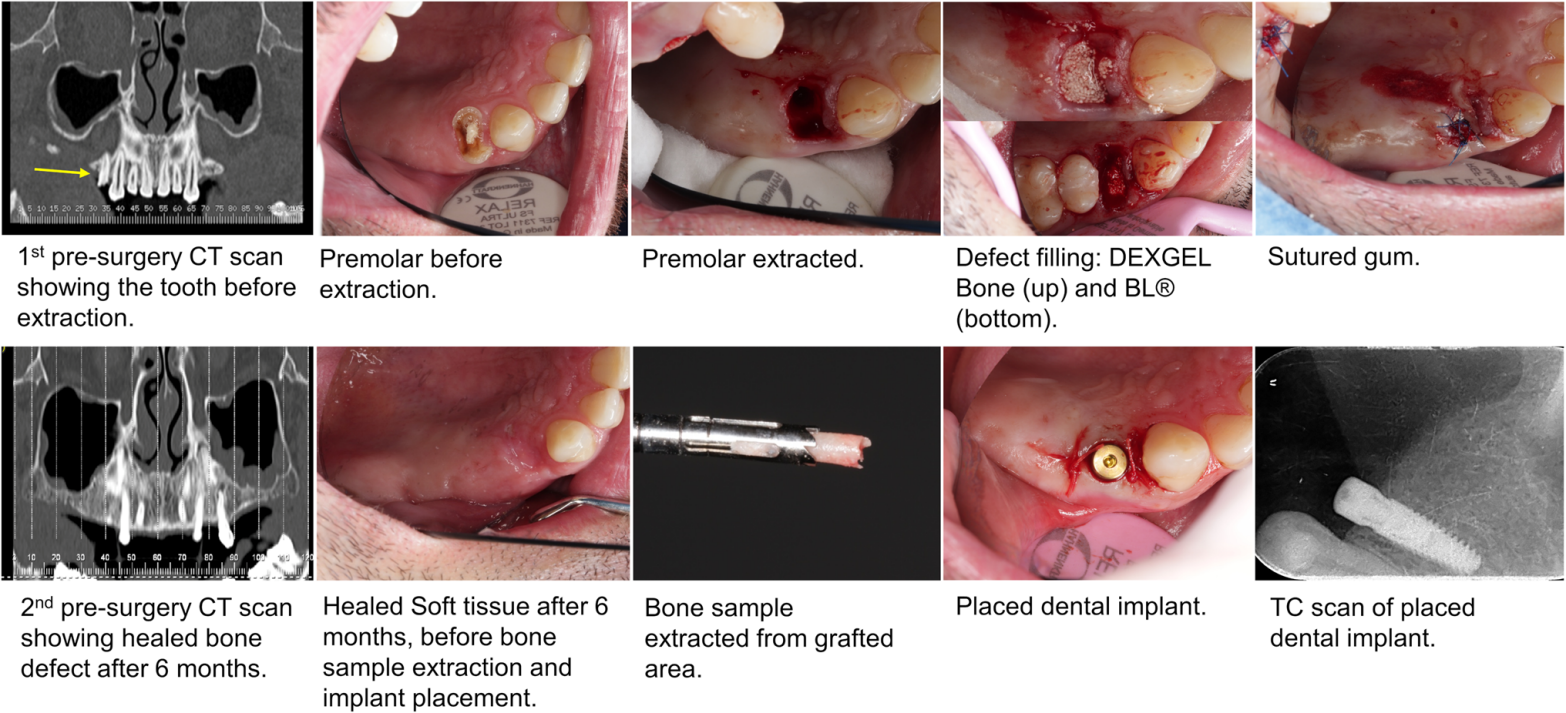
Clinical images illustrating the procedures sequentially ordered from tooth extraction to dental implant placement

Six months after the extraction, another CT and X-ray were performed to compare with the initial ones. The patient was anesthetized, and the crestal incision was made. A bone sample for histologic analysis purposes was taken with a surgical trephine drill of 3-mm diameter and 10-mm length used vertically in an occlusal-apical direction. After this step, titanium implants were finally placed according to the manufacturer (conical dental implant C1, MIS Implants Technologies Ltd., NC, USA). The wound was closed with a pedicle rotated soft tissue graft from the palate, sutured with a 4–0-thickness PROLENE™ polypropylene suture (Ethicon®, Johnson & Johnson, NJ, USA), and another radiograph was performed. The sequence of major events for a DEXGEL Bone group volunteer is depicted in Fig. 3.

### Histological analysis

Histomorphometric analysis was performed at the Hard Tissue Laboratory, Faculty of Medicine, University of Coimbra, Portugal. Bone biopsy samples measuring 6 to 8 mm in length were fixed with 10% formaldehyde (Panreac, Barcelona, Spain) solution buffered at pH 7.4 and stored at 4 °C, until histology analysis of non-decalcified hard tissues with the high-precision Exakt® system (Exakt Technologies, OK, USA). The blocks were sectioned longitudinally in 50–100-μm-thick slices, then stained with toluidine blue and examined with a light microscope (Nikon® Eclipse E600, Tokyo, Japan). The new bone formation, the remaining biomaterial, and the marrow space were quantified in percentages using Bioquant® (Image Analysis Corporation, Nashville, TN).

### Computer tomography

To examine alveolar preservation, CT scans were performed at two time-points: (1) previous to extraction) and (2) after 6 months of biomaterial implantation. Based on an axial section of the upper jaw, the respective curvature was traced, over which radial sections perpendicular to it were obtained, with 1-mm-thick and 1-mm intervals. Also, 1-mm-thick panoramic representations were obtained, one according to the curve mentioned above and also in the palatal and in the vestibular direction with 2-mm intervals.

### Implant Stability Quotient

An Implant Stability Quotient (ISQ) measurement unit was obtained by an advanced non-invasive technique based on resonance frequency analysis, RFA (Penguin RFA, Göteborg, Sweden), commonly used to monitor implant stability. Briefly, a disposable MulTipeg™ (metal transducer with a magnet top) was attached to the implant and magnetically stimulated to vibrate at a micro scale by a handheld probe put closer by 2–4 mm towards the MulTipeg™ top. Then the resonance frequency, i.e., the frequency with the strongest vibration, is measured in a few seconds. Readings were performed from the buccal-lingual and mesial-distal directions. This way, the stiffness of the implant–bone interface was measured and expressed as an ISQ dimensionless value within a scale from 1 to 100, in which the higher the ISQ, the lower the micromotion and the more stable the implant is. Degree of stability can be classified as low for an ISQ < 60, high for an ISQ > 70, and as medium for values in between. ISQ is an objective standard measure reflecting the degree of stability.

### Statistical analysis

In this study, histomorphometry, bone density, bone volume, and primary stability of the implant were compared between test and control groups. Statistical differences in the percentage were assessed by unpaired Student’s *t*-test, and a value of *p* < 0.05 (*) was considered to be significant. Data is presented as mean ± confidence interval (*n* = 6). The normal distribution of the data was assessed by the Shapiro–Wilk test. The analysis was performed using Prism GraphPad 8.02 software® (GraphPad Software, La Jolla, CA, USA). Effect size was measured using Hedges’ *g* formula [36].

## Results

**Fig. 4:**
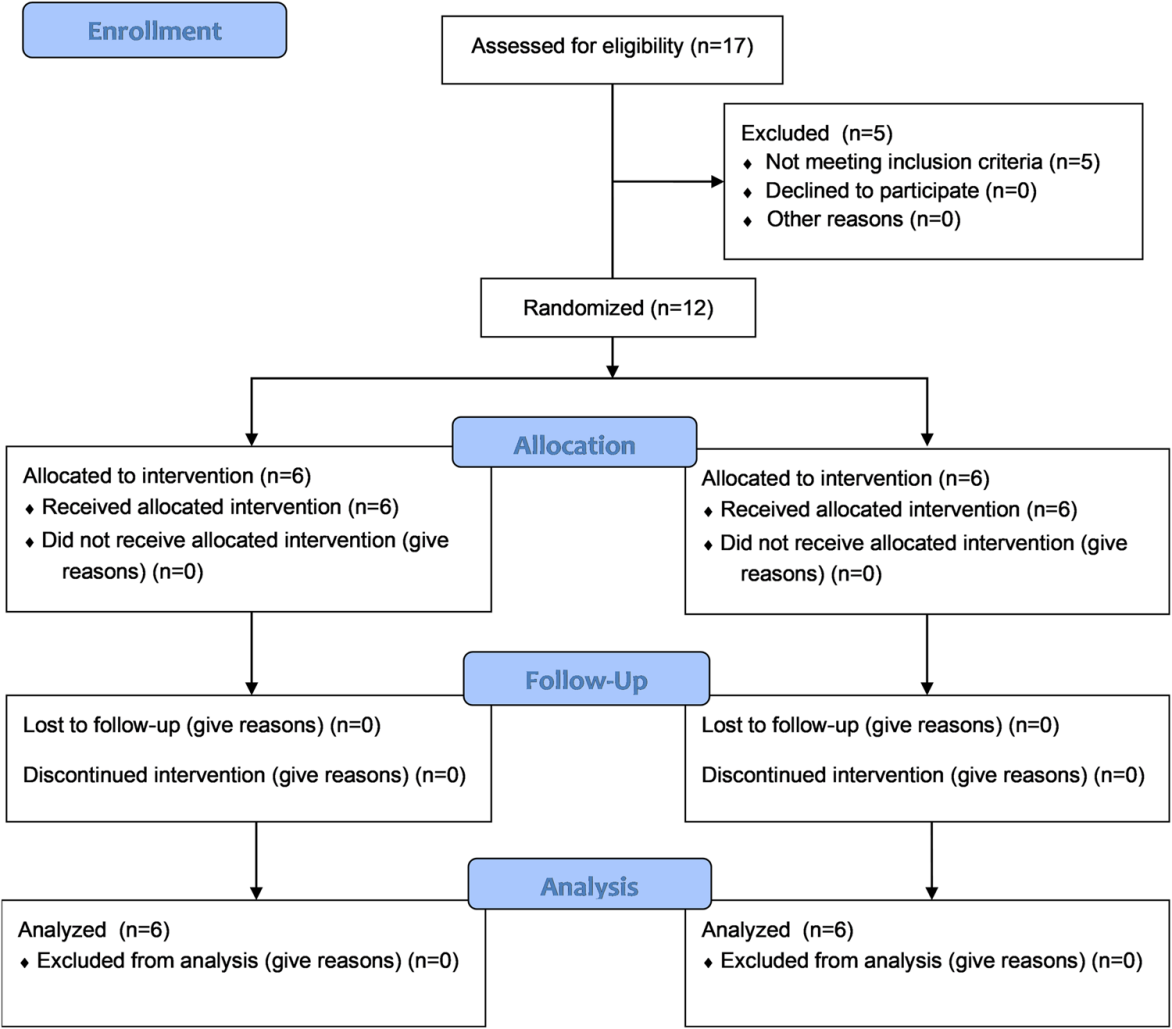
CONSORT flow diagram of this study

The primary endpoint of this study aimed at achieving a volume of bone formed after 6 months equal to the initial post-extraction alveolar volume. As a safety endpoint was the absence of infection and material exposure. A total of 17 volunteers were assessed for eligibility, out of which 5 were excluded for not meeting inclusion criteria described in Table 2. Twelve male and female adults (above 18 years) were randomly assigned into 2 groups of 6 patients each (Table 3). None of the patients discontinued the intervention at any stage of the study (see the CONSORT flow diagram in Fig. 4).

### Clinical analysis

One of the aims of this study included evaluating the capability of DEXGEL Bone to mold into the defect and stabilize BL® granules within it. The device fitted and completely filled the alveolar socket, without leakage of the granules (Fig. 3). DEXGEL Bone displayed improved handling compared to BL® free granules, both during insertion into alveolus and sculpting, operations that were simple to perform and well tolerated by volunteers.

This study was carried out with no signs of local or systemic complications or infection to any volunteer. Patients experienced the usual discomfort associated with oral surgery, with no correlation with grafting material. The sequence of events from exodontia to dental implant placement is shown in Fig. 3. Six months post-grafting, a 2 mm × 10 mm bone sample was collected, and both DEXGEL Bone and BL® groups exhibited suitable bone density for dental implant placement in alveolus. At this stage, the polymeric matrix had already been reabsorbed and only BL® particulate remains were visible.

### Histological and histomorphometric analysis

**Fig. 5:**
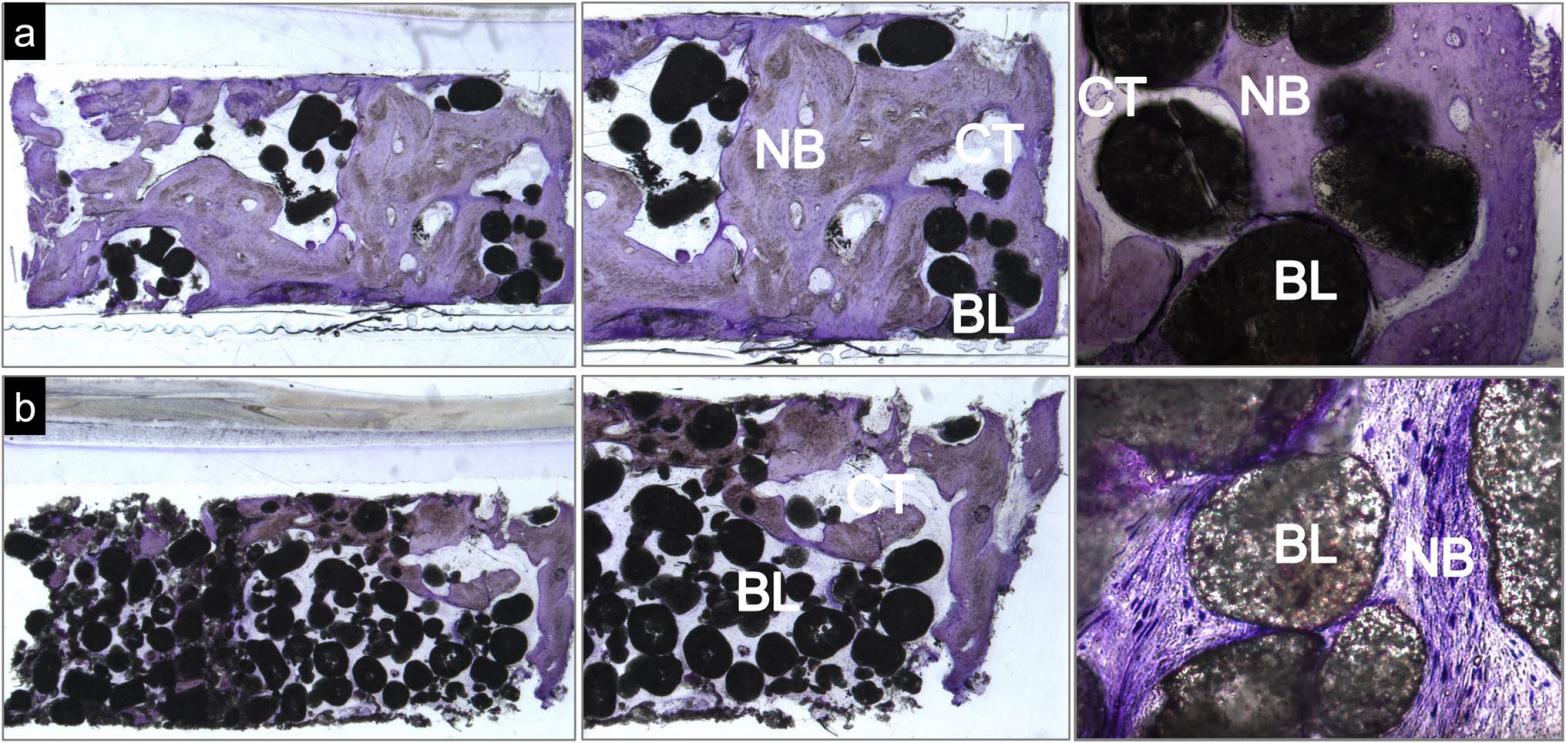
Toluidine blue–stained representative photomicrographs of the grafted site 6 months post-implantation: **a** DEXGEL Bone group and **b** BL® group. NB, new bone; BL, BL® remains; CT, connective tissue. Magnification from left to right: **a** 1 × , 3 × , and 10 × ; **b** 1 × , 3 × , and 20 ×

A qualitative evaluation of the bone tissue collected 6 months after implantation was performed by observation of toluidine blue–stained slides. Figure 5 shows the representative photomicrographs of the samples collected from each group.

Remains of the bone substitute were found in both conditions, in higher amounts in the BL® group. There were no signs of hydrogel after 6 months. New bone was formed on the surfaces and within granules, interspersed with connective tissue. The amount of new bone is notoriously higher in the DEXGEL Bone group. As an important indicator of biocompatibility, no signs of adverse inflammatory reaction were evidenced in both groups. DEXGEL Bone seems to induce a faster new bone regeneration, less unfilled areas being noticed in the histological analysis.

**Fig. 6:**
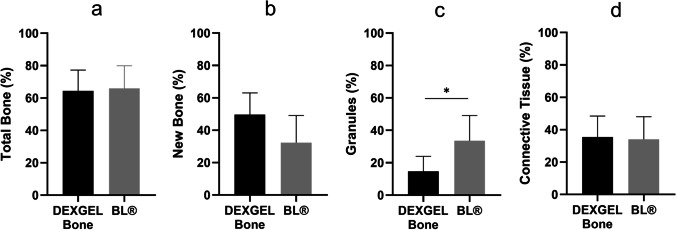
Quantitative histomorphometric results of DEXGEL Bone and Bonelike by Biosckin® (BL®) conditions: total bone (**a**), new bone formation (**b**), reminiscent BL® granules (**c**), and connective tissue (**d**) 6 months after implantation. Statistical analysis was performed using Student’s *t*-test (*p < 0.05). Results are shown as mean percentages ± confidence interval (*n* = 6). Total bone refers to the sum of new bone with granules

The collected bone sample was also analyzed quantitatively with respect to new bone formation, biomaterial remains, and connective tissue (Fig. 6). The use of DEXGEL Bone is considered effective since the total bone volume was similar to that of the BL® group (Fig. 6a). DEXGEL Bone might even be beneficial for bone regeneration since a mean of 49.7% of new bone was reached compared to 32.4% in the BL® group, although the difference is not statistically significant (Fig. 6b). Higher new bone ingrowth was accompanied by more extensive granule resorption (Fig. 6c). Connective tissue ingrowth was similar in both groups, 34.0% and 35.6%, respectively (Fig. 6d). Overall, DEXGEL accelerated BL® reabsorption without compromising (or even stimulating) faster bone growth.

### Bone density and volume

The main application of CT is the preoperative assessment of bone volume and density, providing an important tool for decision-making. Bone density is an important indicator of bone quality for a proper mechanical implant anchorage. Density (HU, Hounsfield units) was analyzed 6 months after biomaterial implantation, prior to implant placement. Five grades were stablished by Misch [37] to classify and distinguish bone from the highest to the lowest density: D1 (> 1250 HU), D2 (850 to 1250 HU), D3 (350 to 850 HU), D4 (150 to 350 HU), and D5 (< 150 HU) (Table 2 SI). The DEXGEL Bone group resulted in a slightly lower density (924), compared to BL® (1114), although with no statistical significance for a *p* < 0.05 (Table 5). The mean value of both groups fall into the D2 grade, characterized by porous cortical bone with coarse trabeculae, typically found in the anterior maxilla and the midpalatal region, consistent with the pre-molar area [38]. D2 classification is indicative of a high strength bone.

**Table 5:** Bone density (HU) of the DEXGEL Bone and BL® groups, for clinician interpretation. Results are shown as mean ± confidence interval (*n* = 6). The differences in bone volume and time-point between the DEXGEL Bone (test) and Bonelike by Biosckin® (BL®, control) groups were analyzed using Student’s *t*-test (*p* < 0.05)

	DEXGEL Bone	BL®
Mean (HU)	924	1114
Standard deviation	168.44	125.00
Range	721–1145	997–1267
Bone quality (Misch [37])	D2	D2
*p* value	0.0516	

*BL®* Bonelike by Biosckin®, *HU* Hounsfield units

**Fig. 7:**
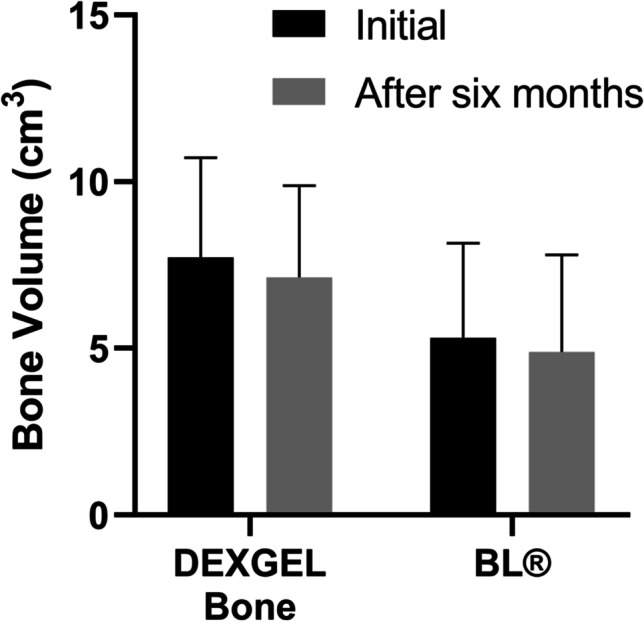
Quantitative bone volume results (cm.^3^) obtained from computer tomography (CT) analysis, at the implantation time and after 6 months. Results are shown as mean percentages ± confidence interval (*n* = 6). The differences in bone volume and time-point between the DEXGEL Bone (test) and Bonelike by Biosckin® (BL®, control) groups were analyzed using Student’s *t*-test (*p < 0.05)

To examine alveolar preservation, initial (post-extraction) bone volume was compared by CT scan to that obtained 6 months after implantation (Fig. 7). Both groups showed a reduction after 6 months, from 7.73 ± 2.99 to 7.13 ± 2.76 cm^3^ in the DEXGEL Bone group, and from 5.32 ± 2.84 to 4.89 ± 2.91 cm^3^ in the BL® group, corresponding to a variation mean of − 7.91% and − 9.84%, respectively, although without statistical significance. Nevertheless, results were more favorable to the DEXGEL Bone group. The addition of hydrogel, therefore, did not compromise bone volume formation.

### Primary stability of the implant

**Fig. 8:**
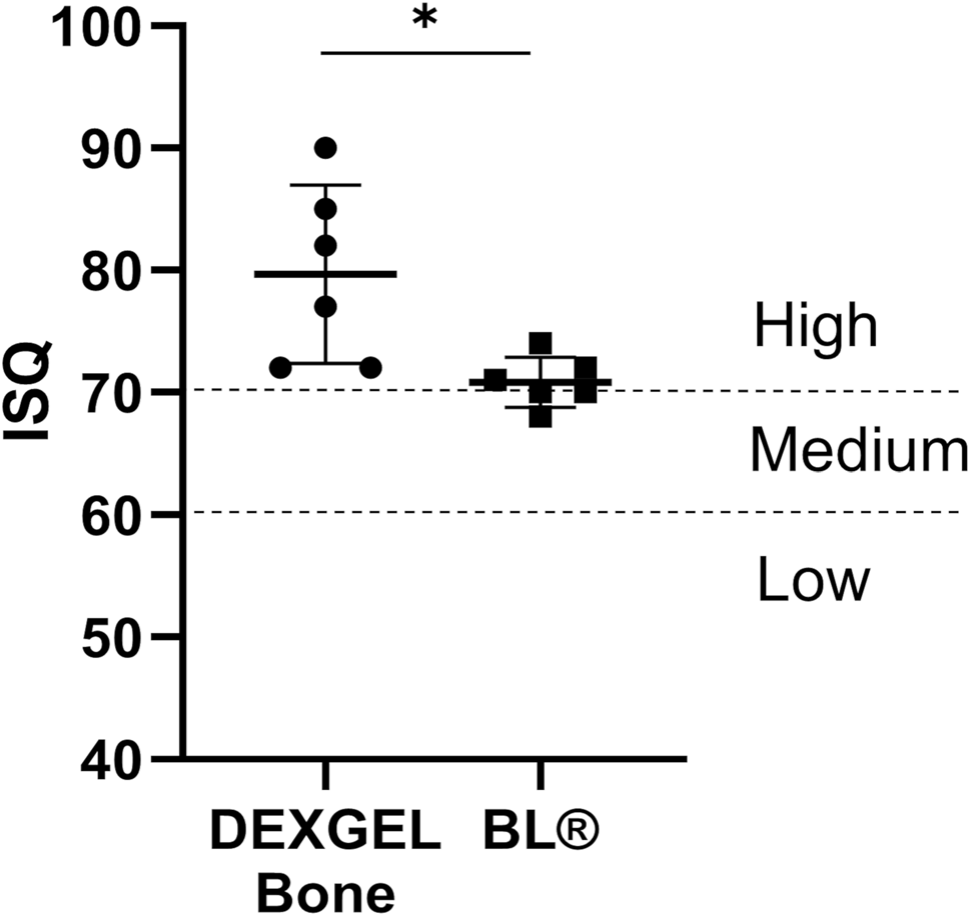
Implant Stability Quotient (ISQ) results of the DEXGEL Bone and Bonelike® (BL®) groups, upon dental implant placement. Statistical analysis was performed using Student’s *t*-test (*p < 0.05). Results are shown as mean values ± confidence interval (*n* = 6)

ISQ analysis was used to evaluate primary stability, upon implant placement. The mean ISQ value for the DEXGEL Bone group was 79.7 ± 7.3 and 70.8 ± 2.0 for the BL® group (Fig. 8). Though the mean values obtained for both groups fall into a high-stability classification (ISQ > 70), the DEXGEL Bone group showed statistically superior stability (*p* = 0.0170). In the DEXGEL Bone group, three out of 6 patients exhibited individual ISQ values from 82 to 90 and none below 70, while in the BL® group no value above 74 was recorded, one individual presenting a value of 68. Therefore, the addition of the hydrogel matrix to BL® granules did not compromise bone quality in terms of stability, apparently being even beneficial for the subsequent restauration. Nevertheless, immediate implant loading with provisional prostheses was not performed in both groups to avoid mechanical pressure. Ten weeks post-implantation, silicon impressions were taken, and at week 13, implants were loaded with the final restoration. No complications were detected during follow-up.

Effect size is a quantitative measure of the magnitude of the experimental effect. Measures of 0.2, 0.5, and 0.8 are considered small, medium, and large effect sizes, respectively. Effect size measures below 0.2 may express a negligible difference between two groups, even if statistically significant. Table 6 shows the effect size values that show if the treatment had a small, medium, or large effect on the designated outcomes. Compared to the BL® group, DEXGEL Bone exhibited a loss associated with a small effect size in total bone (*g* =  − 0.11) and large in granules (*g* =  − 1.36) and density (*g* =  − 1.18) outcomes, as expressed by the negative Hedges’ *g* value. Effect size measures were also small in the primary outcome for DEXGEL Bone (*g* =  − 0.21) and for BL® (*g* =  − 0.15), suggesting that the difference between the initial bone volume and the same after 6 months is unimportant, as also indicated by *p* > 0.05. Positive Hedges’ *g* expresses a gain associated with a large effect size in respect to new bone (*g* = 1.05) and ISQ (*g* = 1.52) analyses, but small in connective tissue (*g* = 0.11).

**Table 6:** Summary of mean and *p* value results, assessing the effect size of each outcome by Hedges’* g* calculations

Outcome	Mean DEXGEL Bone (test)	Mean BL® (control)	*p* value^b^	Hedges’*g*^a^
Total bone (%)	64.4	66.0	0.847	− 0.11
New bone (%)	49.7	32.4	0.076	1.05
Granules (%)	14.7	33.6	0.029	− 1.36
Connective tissue (%)	35.6	34.0	0.847	0.11
Density (HU)	924	1114	0.052	− 1.18
ISQ	79.7	70.8	0.017	1.52
	Mean (after 6 months)	Mean (initial)	*p* value^b^	Hedges’ *g*^c^
Bone volume (cm^3^) for DEXGEL Bone	7.13	7.73	0.722	0.19
Bone volume (cm^3^) for BL®	4.89	5.32	0.803	0.14

^a^The effect size was estimated by the difference (test–control) in mean change in the specified outcome measurement and represents the gain associated with treatment

^b^Significance probability associated with the Student’s *t*-test performed to compare the means of test and control (*p < 0.05)

^c^The effect size was estimated by the difference (after–initial) in mean change in the specified outcome measurement and represents the gain associated with the 6-month time-point

Despite a non-significant *p*-value, new bone and density outcomes show large effect sizes, being indicative of an advantageous stimulus to the regenerative process provided by DEXGEL Bone. The significant *p*-values of granule quantification and ISQ are reinforced by large effect sizes, and non-significant *p*-values are reinforced by a low effect size for total bone, connective tissue, and bone volume after 6 months.

## Discussion

This is the first-in-human study of DEXGEL Bone on safety and efficacy. No local or systemic complications or infections were identified, and none of the participants were excluded from the study. A synthetic bone substitute, BL® (control), was compared to its hydrogel-reinforced version, DEXGEL Bone (test), in the preservation of the alveolar ridge dimensions following tooth extraction. Six months after exodonty and grafting, CT scans showed healed bone defects in both groups, suitable for bone sample collection and dental implant placement. The implants were eventually loaded with prostheses, generally 13 weeks post-implantation. Major outcomes of this study include handling properties, safety validation, assessment of bone quantity and quality, and primary stability of implant.

From a commercial point of view, an extended shelf-life is important for any product. However, extended stability studies on hydrogels for biomedical applications are scarce in literature. The reticulation of DEXGEL is driven by covalent interactions between ODEX aldehydes and ADH amines, and gelation time can be manipulated to vary between a few seconds to several hours, depending on ODEX and ADH concentrations or on the oxidation degree [12]. Thus, a reduction in the content of reactive groups induced by any structural modifications, such as degradation, would interfere with the gelation period. We performed a gelation study using sterilized ODEX and ADH solutions stored at 4 °C for up to 3 years. The term “gelation” here refers to the moment when it was no longer possible to pipette the hydrogel — which is the relevant form for clinical handling — irrespective of crosslinking extent reactions. The gelling time (30 s) of an ODEX mixture, prepared with fresh ADH, did not suffer any changes up to 3 years, demonstrating an excellent stability (Table 1 SI).

Stability and conformability are of particular importance, to avoid the release or migration of particles, assess difficult sites, mold to the defect, and provide a reproducible and homogeneous mixing procedure convenient to the surgeon. Synthetic bone substitutes are now available in different forms, though with some shortcomings on handling properties. For instance, granules can migrate out of the defect during and after surgery; microporous blocks may be difficult to fit within the defect; and cement paste might set too fast and, similar to putty, can be poorly injectable [39]. The addition of DEXGEL to BL® improved granule cohesivity, by turning it into a moldable paste-like material, easy to administer with a syringe in the maxillary alveolar socket of the pre-molar area, avoiding granule leakage until wound closure. DEXGEL Bone is neither too liquid nor to viscous; therefore, extrusion force was suitable as to provide an easy control of biomaterial outflow from the syringe opening [18]. BL® granules (mixed with autologous blood), on the other hand, had to be administered with a spatula and were prone to leakage during socket loading. Since DEXGEL is produced separately from the bone substitute, combinations with other commercially available grafting materials can be considered. DEXGEL is able to pass through a needle (injectability), with the diameter of the opening being limited by the size of the particles to which it is combined. The association of this HG to a particulate bone substitute would enable further loading of molecules with bone healing or antimicrobial properties.

Clinically, no complications were observed and all participants healed normally. Histologically, both test and control groups showed good integration of grafting material into newly formed bone and were biologically compatible with the host tissues, showing no signs of adverse reaction. Photomicrographs confirmed the formation of vascularized mature bone and soft tissue matrix, confirming the osteoconductive character of both test and control groups. A considerably lower number of granules were visible in the test group, interspaced by a thicker new bone formation, as compared to the control group. In accordance with histologic observations, histomorphometric examination showed DEXGEL-accelerated BL® reabsorption without compromising total bone growth, as we have previously shown in the regeneration of critical-sized defects in a goat model [17]. The higher rate of BL® granule resorption may have opened up space for more new bone ingrowth in the DEXGEL Bone group. The balance between resorption of a scaffold and its replacement by new bone formation is a key factor to shorten bone healing time. The addition of DEXGEL to BL® apparently optimized this balance. This higher resorption effect may be explained by the acidic character provided by aldehyde-bearing ODEX. The pH of the ODEX solution around 3.0 increases to 4.7 after adding BL® granules in the first step of biomaterial preparation. The subsequent addition of ADH solution (pH = 7.4) further increases pH of the final formulation to 5.2. After implantation, the more soluble TCP phase is prone to a faster resorption rate than HA [40], which can be considerably accelerated by pH acidification [41].

Ideally, scaffold materials should degrade synchronously as new bone ingrowth takes place, without loss of mechanical support. As the bone regeneration process takes place, BL® is also resorbed in a slow and controlled manner, contributing to the natural remodeling of the bone [42]. BL® is composed by a modified HA (≥ 50%) matrix, TCP, and ionic species commonly found in human bone, i.e., magnesium, sodium, and fluor (≤ 50%). The addition of the TCP phase confers a degradability character to the non-degradable hydroxyapatite (HA) [40, 43]. The TCP resorption mechanism is essentially cell-mediated [39, 44]: bone remodeling cells, osteoclasts, release hydrochloric acid at the material surface, inducing calcium phosphate dissolution by acidification [45]. Polymer-based bone graft substitutes are resorbed by hydrolysis [39]. The reversibility nature of ADH and ODEX crosslinking [14] gradually releases 1–4 α-linked glucose dextrin, which can be enzymatically decomposed by blood α-amylases [46]. Dextrin and its degradation products can be metabolized or undergo renal elimination, owing to its low molecular weight (~ 2 kDa for Tackidex® B 167) below the renal filtration limit range (~ 30–50 kDa) [46, 47]. Overall, DEXGEL Bone resorption can occur by several ways such as dissolution, cell-mediated dissolution, hydrolysis, and enzymatic decomposition.

Despite the favorable biodegradability, the use of polymeric bone grafters is restricted by limitations related to acidic degradation products that may accelerate implant deterioration and induce inflammatory reactions with negative implications for tissue repair [48, 49]. In this study, DEXGEL was not detected 6 months post-treatment prior to implant placement, neither has it ever been detected in previous pre-clinical studies from three weeks on [16–18]. Interference with implants would not therefore be a concern. The HG improved the granule cohesivity and ease of handling at the time of administration, as intended (injectability and mouldability), then being fully resorbed rapidly, opening up space for more new bone growth.

The success of dental implant placement relies on both the alveolar bone volume and density. The first may allow implant placement in the three-dimensionally correct position or, on the contrary, prevent its placement due to inadequate bone dimensions. In this latter case, a second-regeneration treatment simultaneously with implant placement may be considered. The second may essentially influence the primary stability of the implant, its osseointegration, and the timing of prostheses loading. Human studies on dimensional changes of undisturbed alveolar natural healing have reported horizontal bone loss of 29–63% and vertical bone loss of 11–22% at 6 months post-extraction [50]. In this work, we report bone volume variation means of − 7.91% and − 9.84% for the test and control groups, respectively. The addition of HG showed no constraints on this parameter. Bone density could be defined as the amount of bone filling within a certain bone volume, being indicative of bone ability to ensure proper mechanical attachment of an implant [51]. Indeed, primary retention is achieved by mechanical means rather than through osseointegration. Implants on low-density bone are more likely to fail [52]. In our study, DEXGEL Bone did not compromise the regeneration process, evidencing a good-quality bone with a density classification of D2 (Misch [37]), the typical structural conformation of a common pre-molar tooth region [38]. With respect to strength, D1 and D4 bones are spaced by a tenfold difference, in which, if converted to a scale of 1 to 10 from the least to the greatest strength, D1 could be seen as a 9 or 10, D2 a 7 or 8, D3 a 3 or 4, and D4 a 1 or 2 [37]. Implant failure has been reported as 5% in D1 bone, 2.2% in D2, 13.6% in D3 and 19.2% in D4 [53]. While a healing period of 3 to 4 months would be adequate for D1 and D2 before implant loading, 5 to 6 months would be required for D3 and D4.

ISQ can ultimately determine whether or not the implant will withstand the impact of a provisional or a final restauration, and the subsequent mechanical impact from masticatory forces when integrated in the overall dentition. ISQ is, therefore, important for decision-making during implant treatment and follow-up [54]. Although both conditions occasioned an excellent ISQ mean (> 70) at implant placement, administration of DEXGEL Bone generally resulted in a higher primary stability of dental implant, indicating that the presence of DEXGEL did not compromise immediate osseointegration, being even advantageous. In these cases, immediate implant loading is accepted. A higher ISQ value may be explained by the higher tendency for more new bone ingrowth, as a positive linear correlation between both has been reported [51]. ISQ values were high even for hydroxyapatites grafted sites [55]. ISQ changes over time are reported in many studies, describing an initial decline lasting from up to the first 2 weeks to 3 months, followed by a gradual increase up to higher or similar values to the original one [56]. Therefore, although ISQ evaluation taken later at restoration placement (secondary stability) seems more important for success predictability than at implant placement (primary stability) [57, 58], initial ISQ values of this study may nevertheless be indicative of implant clinical success. These results further indicate that DEXGEL Bone would possibly enable the earlier placement of a final prosthesis, shortening the conventional period of 3 to 6 months, improving the life quality of the patient. This study also confirms that changing the physicochemical properties of a bone substitute material can influence implant stability.

In this study, we used the 250–500-µm spherical BL® which has shown to induce a slightly faster bone regeneration than 500–1000 µm [42]. The presence of adequate pore dimension in BL® favors osteointegration, osteoconduction, and degradation, allowing bone ingrowth in the interspaces, as it enables blood vessels and cell infiltration, exchange of proteins and nutrients, and waste clearance. Overall, BL® provides an ideal environment for bone adhesion, cell proliferation, and differentiation; has a slow and controlled resorption; and contributes to natural bone remodeling. DEXGEL provides an easy and effective filling of bone defects according to its irregularities, given its excellent handling and molding properties. It was rapidly resorbed and accelerated BL® resorption as well, freeing up space that favored new bone ingrowth, without compromising mechanical support. The small population size is a limitation of this study, which however was adequate to prove the safety of this HG in its first contact with humans. Larger samples can now be considered in future studies. The present study validates DEXGEL Bone suitability for oral rehabilitation with endosseous implants, although other clinical scenarios may be considered.

## Conclusion

DEXGEL Bone is a moldable, easy-to-apply bone regeneration optimized technology that naturally stimulates bone formation. The addition of DEXGEL to BL® granules provided the biomaterial with injectability without compromising bone volume nor density. DEXGEL Bone even showed a tendency for more new bone formation and maximized primary stability of the dental implant, which have reported a positive correlation. A clinical benefit is therefore achieved: improved granule cohesivity, easier handling, delivering (injectability), and sculpting of the biomaterial within the alveolar socket. DEXGEL Bone did not cause pain, discomfort, or infections, being effective in alveolar ridge preservation. DEXGEL is easy to produce, is cost-effective, has a long-shelf life, and can provide benefits to commercially available grafting materials in terms of injectability, moldability, and clinical performance. This in situ gelling HG further provides a platform for the entrapment of specific therapeutic agents to meet other clinical scenarios. Some conclusions of this study may be limited by a small population size, which however was adequate for the first human safety assessment. Now with safety validation, larger populations could be considered in the future.

## Supplementary Information

Below is the link to the electronic supplementary material.

Supplementary file1 (DOCX 288 KB)

## Data Availability

Data available within the article or its supplementary materials.
