# Supplementary material for: Randomized clinical study of injectable dextrin-based hydrogel as a carrier of a synthetic bone substitute
Source: Clin Oral Investig. 2023 Jan 28;27(3):979–94. doi: 10.1007/s00784-023-04868-9 (PMC9985577; doi:10.1007/s00784-023-04868-9)
Supplement: Supplementary file 1 — Supplementary file1 (DOCX 288 KB) [file 784_2023_4868_MOESM1_ESM.docx]

# Supplementary Materials

**Randomized Clinical Study of Injectable Dextrin-based Hydrogel as a Carrier of a Synthetic Bone Substitute**

Alexandra Machado, MSc^1,2^, Isabel Pereira, PhD^1,2^, Filomena Costa, PhD^1,2^, Ana Brandão, PhD^3^, José Eduardo Pereira, PhD^4,5^, Ana Colette Maurício, PhD^6,7^, José Domingos Santos, PhD^8^, Inês Amaro, MSc^9^, Rui Falacho, PhD^10^, Rui Coelho, PhD^11^, Nuno Cruz, PhD^12^, Miguel Gama, PhD^1,2*^

^1^ CEB, Centre of Biological Engineering, University of Minho, 4710-057 Braga, Portugal.

^2^ LABBELS, Associate Laboratory, Braga, Guimarães, Portugal

^3^ Biosckin, Molecular and Cell Therapies S.A., TecMaia, Rua Engenheiro Frederico Ulrich 2650, 4470-605 Maia, Portugal.

^4^ CECAV, Animal and Veterinary Research Centre, University of Trás-os-Montes and Alto Douro, Vila Real 5001-801, Portugal.

^5^ Department of Veterinary Sciences, University of Trás-os-Montes and Alto Douro, Vila Real 5001-801, Portugal.

^6^ Departamento de Clínicas Veterinárias, Instituto de Ciências Biomédicas de Abel Salazar (ICBAS), Universidade do Porto (UP), Rua de Jorge Viterbo Ferreira, n° 228, Porto 4050-313, Portugal.

^7^ Centro de Estudos de Ciência Animal (CECA), Instituto de Ciências, Tecnologias e Agroambiente da Universidade do Porto (ICETA), Rua D. Manuel II, Apartado 55142, Porto 4051-401, Portugal.

^8^ REQUIMTE/LAQV, Departamento de Engenharia Metalúrgica e Materiais, Faculdade de Engenharia, Universidade do Porto, Rua Dr. Roberto Frias, Porto 4200-495, Portugal.

^9^ Institute of Integrated Clinical Practice, Faculty of Medicine, University of Coimbra, Coimbra 3004-504, Portugal.

^10^ Institute of Oral Implantology and Prosthodontics, Faculty of Medicine, University of Coimbra, Coimbra 3004-504, Portugal.

^11^ RESDEVMED, Unipessoal Lda., Travessa do Navega, 436 C, Ovar 3885-183, Portugal.

^12^ Faculty of Dentistry, Universitat Internacional de Catalunya, Barcelona 08017, Spain.

^*^Corresponding author: Miguel Gama, CEB, Centre of Biological Engineering, University of Minho, Campus de Gualtar, 4715-057 Braga, Portugal. Tel. (office): +351 253 604 418. E-mail address: fmgama@deb.uminho.pt (M. Gama). ORCID ID: 0000-0002-5655-0015.


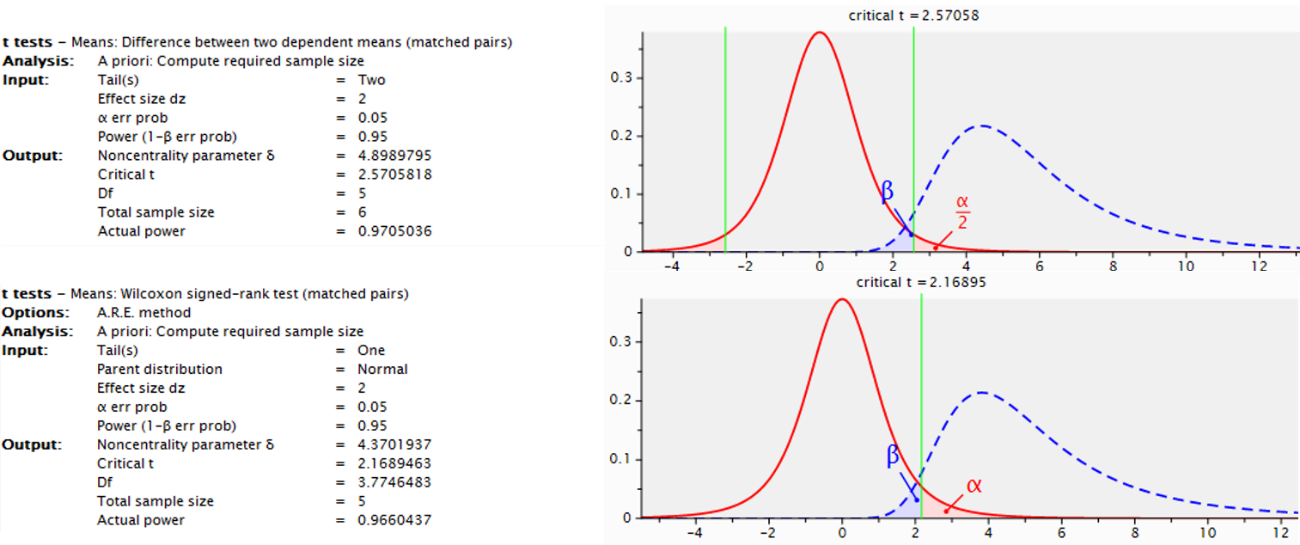


**Fig.1 SI** Study designed for a two-tailed analysis with Student's t-test or Wilcoxon signed rank test (depending on the normality of the results), with a confidence interval (statistical power) of 95% (1-β=0.95). G*Power 3.1.9.2 software was used to define sample size.

**Table 1 SI.** Gelation time of HG upon mixture of ODEX and ADH, expressed in seconds (n=3). After production and sterilization, ODEX (30% w/v) and ADH (3.76% w/v) solutions were separately kept at 4⁰C and a gelling test was performed at different time-points by mixing both in a proportion of 7:3 volume, respectively. Freshly prepared ADH was also used for crosslinking in each time-point with the ODEX stored over time.

| Mixture (7:3 ratio) | Initial | 3 months | 1 year | 3 years |
| --- | --- | --- | --- | --- |
| ODEX + ADH | 30 ± 2 sec | 30 ± 2 sec | 30 ± 2 sec | - |
| ODEX + fresh ADH | 30 ± 2 sec | 30 ± 2 sec | 30 ± 2 sec | 30 ± 2 sec |

ODEX, oxidized dextrin; ADH, Adipic dihydrazide.

**Table 2 SI.** Bone density classification by Misch [36].

| **Classification** | **Hounsfield Units** | **Description** | **Typical Anatomical location** |
| --- | --- | --- | --- |
| D1 | >1250 | Dense cortical | Anterior mandible |
| D2 | 850-1250 | Thick dense to porous cortical and coarse trabecular | Anterior and posterior mandible, anterior maxila |
| D3 | 350-850 | Thin porous cortical and fine trabecular | Posterior mandible, anterior and posterior maxila |
| D4 | 150-350 | Fine trabecular bone | Posterior maxila |
| D5 | <150 | Poorly mineralized bone |  |
